# Comparative proteomic analysis of regenerative mechanisms in mouse retina to identify markers for neuro-regeneration in glaucoma

**DOI:** 10.1038/s41598-024-72378-z

**Published:** 2024-10-04

**Authors:** Xiaosha Wang, Layla Frühn, Panpan Li, Xin Shi, Nini Wang, Yuan Feng, Julia Prinz, Hanhan Liu, Verena Prokosch

**Affiliations:** 1 Department of Ophthalmology, University of Cologne, Kerpener Str. 62, 50937 Cologne, Germany; 2 Cologne Excellence Cluster on Cellular Stress Responses in Aging-Associated Diseases (CECAD), Faculty of Mathematics and Natural Sciences, University of Cologne, 50931 Cologne, Germany; 3 Department of Ophthalmology, RWTH Aachen University, 52074 Aachen, Germany

**Keywords:** RGC regeneration, Glaucoma, RGC degeneration, Mass spectrometry, Proteomic landscape, Regeneration and repair in the nervous system, Optic nerve diseases

## Abstract

The retina is part of the central nervous system (CNS). Neurons in the CNS and retinal ganglion cells lack the ability to regenerate axons spontaneously after injury. The intrinsic axonal growth regulators, their interaction and roles that enable or inhibit axon growth are still largely unknown. This study endeavored to characterize the molecular characteristics under neurodegenerative and regenerative conditions. Data-Independent Acquisition Mass Spectrometry was used to map the comprehensive proteome of the regenerative retina from 14-day-old mice (Reg-P14) and adult mice after lens injury (Reg-LI) both showing regrowing axons in vitro, untreated adult mice, and retina from adult mice subjected to two weeks of elevated intraocular pressure showing degeneration. A total of 5750 proteins were identified (false discovery rate < 1%). Proteins identified in both Reg-P14 and Reg-LI groups were correlated to thyroid hormone, Notch, Wnt, and VEGF signaling pathways. Common interactors comprising E1A binding protein P300 (EP300), CREB binding protein (CBP), calcium/calmodulin dependent protein kinase II alpha (CaMKIIα) and sirtuin 1 (SIRT1) were found in both Reg-P14 and Reg-LI retinas. Proteins identified in both regenerating and degenerative groups were correlated to thyroid hormone, Notch, mRNA surveillance and measles signaling pathways, along with PD-L1 expression and the PD-1 checkpoint pathway. Common interactors across regenerative and degenerative retinas comprising NF-kappa-B p65 subunit (RELA), RNA-binding protein with serine-rich domain 1 (RNPS1), EP300 and SIN3 transcription regulator family member A (SIN3A). The findings from our study provide the first mapping of regenerative mechanisms across postnatal, mature and degenerative mouse retinas, revealing potential biomarkers that could facilitate neuro-regeneration in glaucoma.

## Introduction

The retina constitutes a part of the central nervous system (CNS) and shares remarkable similarities to the brain. Both retinal and CNS neurons lack the capacity to regenerate axons following injury. Glaucoma, a neurodegenerative disease, shows damage to retinal ganglion cells (RGC) and their axons, ultimately leading to irreversible cell death and vision loss. Whilst enhancing the survival of RGCs is a crucial initial step in the treatment, therapies should also address axonal regeneration. However, regeneration of axons in RGCs is a complex process involving neuronal and non-neuronal changes, all working at cross-purposes, activating signaling pathways, altering the expressions of genes and proteins within the neuron that enable or inhibit axonal growth^1,2^. Despite decades of effort and resources invested in the research of neuroregeneration, scientists are still rather far from comprehensively identifying all intrinsic axonal growth regulators and their collaborative roles.

The ability of RGCs to extend their axons decreases with age and is lost early in development^3^. Juvenile RGCs show spontaneous regeneration under regenerative conditions in vitro. However, even adult RGCs can regrow their axons in experimental conditions, implying that they do not lose their regenerative potential completely^4^. One approach to induce regeneration both in vivo and in vitro is accomplished by lens injury (LI) and optic nerve crush (ONC)^5^. It is known that proteins execute their functions through interactions with other proteins. Protein–protein interaction (PPI) modalities are crucial elements that facilitate intercellular communication and modulate signal transduction pathways. Ultimately, they govern the modulatory actions of both degeneration and regeneration.

Recent advancements in mass spectrometry (MS)-based proteomic techniques, coupled with bioinformatics tools, have facilitated the comprehensive mapping of protein signaling networks across various sample types. Data-independent acquisition mass spectrometry (DIA-MS) is a next-generation proteomic methodology that provides superior reproducibility and sensitivity compared to conventional MS.

This study, building upon our previous studies^6,7^, offers the first comprehensive exploration of the global proteome landscape, unraveling molecular intricacies and signaling pathways pivotal in both degenerative and regenerative processes in rodent retinas. These insights hold translational potential for identifying novel therapeutic targets for glaucoma.

## Results

### EVC induced IOP elevation

**Fig. 1:**
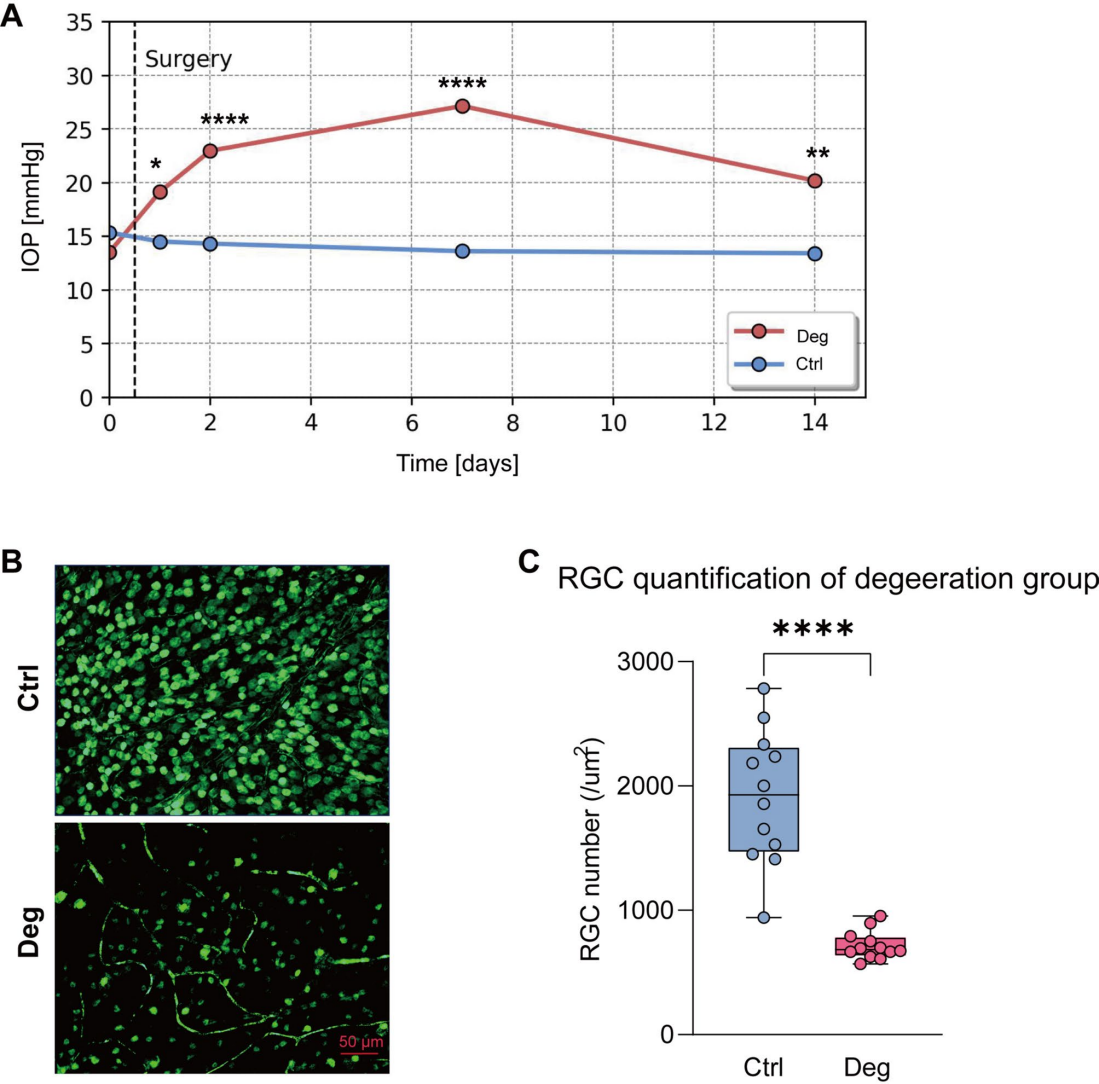
Impact of Episcleral Vein Cauterization (EVC) on Intraocular Pressure (IOP) and Retinal Ganglion Cell (RGC) Density. (**A**) IOP dynamics post- EVC. (**B**) Fluorescence images of Brn3a-positive RGCs in retinal flat-mounts. (**C**) RGC density across control and EVC groups.

Before the EVC surgery in degeneration (Deg) group, IOP levels in eyes designated for EVC were similar to those in control eyes. After the EVC, a significant increase in IOP was observed at day 1 (*p* = 0.012), day 2 (*p* < 0.0001), day 7 (*p* < 0.0001) and day 14 post-intervention (*p* = 0.003) (Fig. 1A). Animals were sacrificed two weeks post-intervention.

### EVC impairs RGC survival

EVC resulted in a significant reduction in RGC density (716.2 ± 114.7 RGC/mm^2^) in versus their age-matched controls (1911.7 ± 534.3 RGC/mm^2^) (Fig. 1B and C).

### P14 and LI induced axon regeneration

**Fig. 2:**
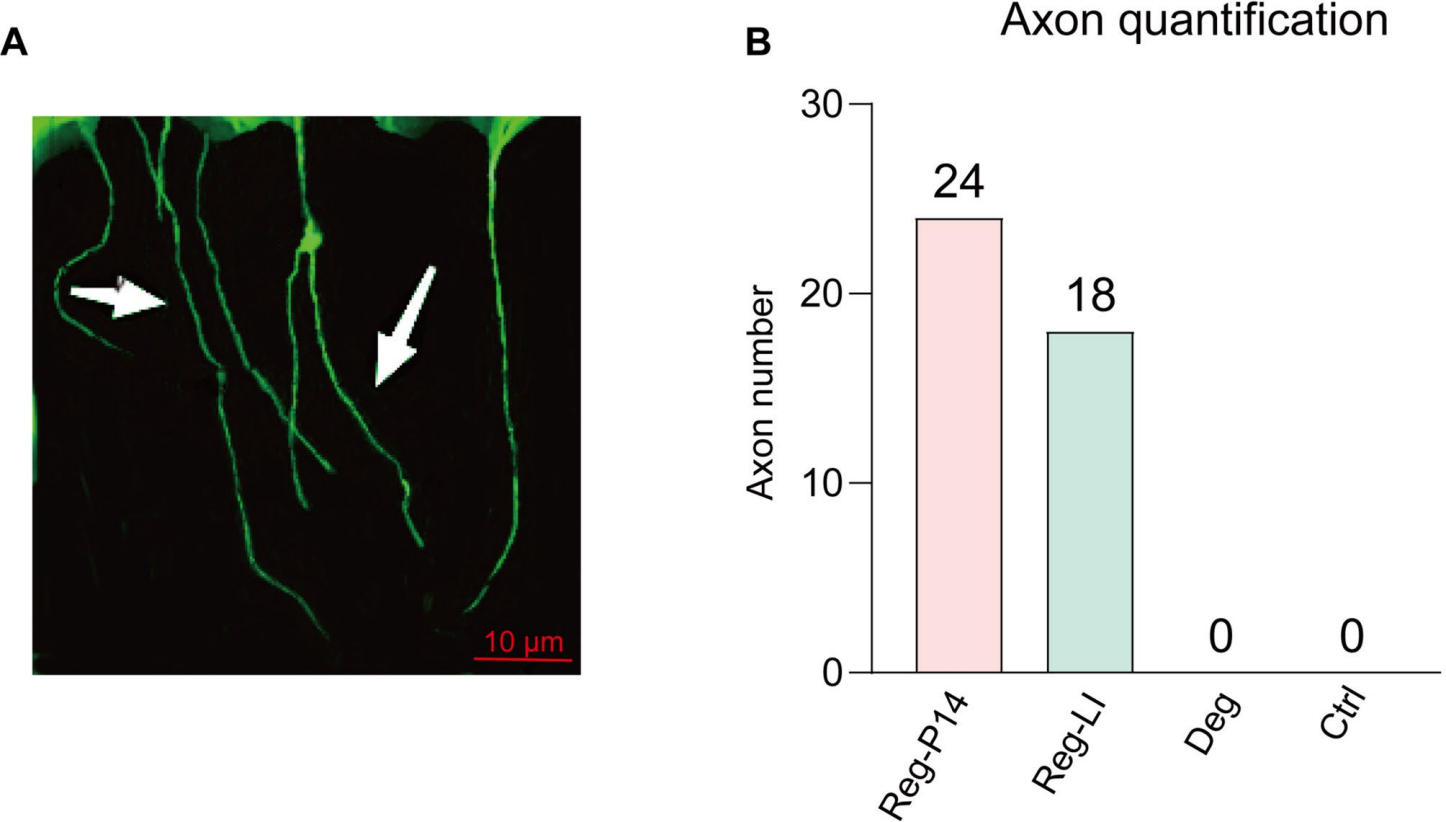
Analysis of Axon Regeneration in Regenerative Postnatal Day 14 (Reg-P14), Lens Injury (Reg-LI) and degeneration (Deg) Groups. (**A**) Axonal extensions from retinal explants in the Reg- P14 group. (**B**) Evaluation and comparison of the regenerated axon numbers across Reg-P14, Reg-LI, Deg and control groups.

On day 7 after incubation in the regenerative condition, retinas extracted from both the Reg-P14 group and the Reg-LI group demonstrated axonal outgrowth (Fig. 2A). A total outgrowth of 24 neurites was counted in the Reg-P14 group and 18 neurites per 1/8th of retina in the Reg-LI group. Explants from control and Deg groups showed no outgrowth of axons (Fig. 2B).

### Proteomic profile

**Fig. 3:**
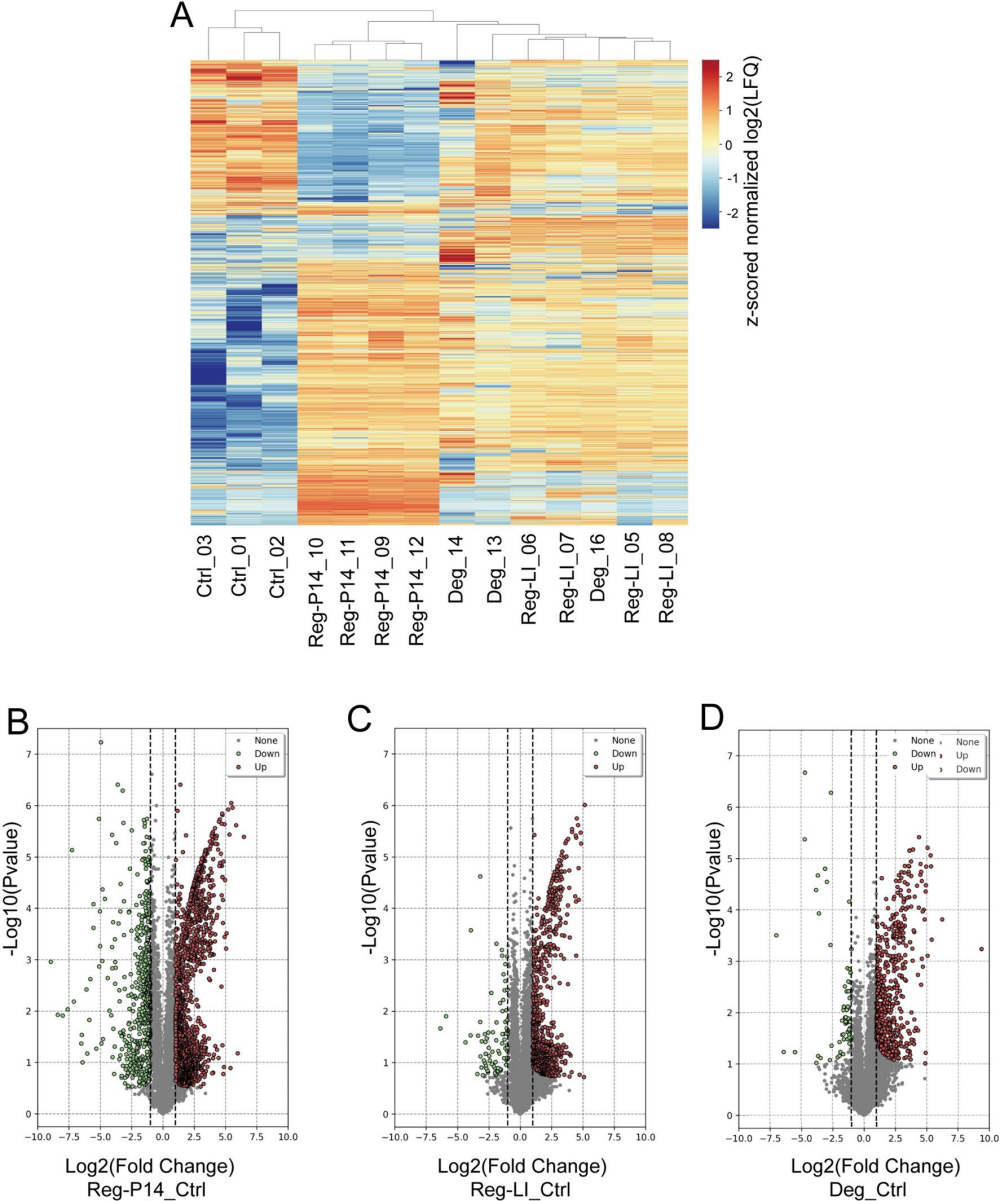
Comprehensive Overview of Whole Proteomic Data. (**A**) Clustered heatmap displaying differential protein expression profiles across four experimental groups: Control (Ctrl), Postnatal Day 14 (Reg-P14), Lens Injury (Reg-LI), and Degeneration (Deg). This visualization delineates patterns of protein abundance variations among the groups. (**B**–**D**) Volcano plots emphasizing proteins differentially expressed in comparison to the control group across three specific conditions: (**A**) Reg-P14 versus Control; (**B**) Reg-LI versus Control; (**C**) Deg versus Control. Statistical significance was established at an adjusted *p* value threshold of ≤ 0.05. Differential expression was defined by a log2 fold change greater than 1 for upregulation or less than − 1 for downregulation. In these plots, red points indicate upregulated proteins and green as downregulated.

The proteomic profile of the retina reveals the presence of 5182 proteins in the retina of the control group, 5671 proteins in the spontaneously regenerative retina from Reg-P14 group, 5631 proteins in the experimentally regenerative retina in Reg-LI group, and 5628 proteins in the degenerative retina in Deg group. The expression profiles of all detected proteins were illustrated using a cluster heatmap (Fig. 3A). When compared to the control group, the experimental groups demonstrated distinct expression patterns. Notably, the Deg and the Reg- LI group exhibited highly similar expression patterns, indicating that the molecular responses or pathological processes involved in experimental regeneration and degeneration may converge on shared mechanisms or pathways.

### DEPs across P14, LI, and Deg groups versus control

Subsequent investigation of these proteins identifies several differentially expressed proteins (DEPs) between control, Reg-P14, Reg-LI and Deg groups. The evaluation is based on the normalized abundances, and proteins were considered differentially expressed if they exhibited either a ≥ twofold upregulation or a ≤ 0.5-fold downregulation, accompanied by adjusted *p* value ≤ 0.05.

In the Reg-P14 group, a total of 1961 DEPs were identified, of which 1412 proteins were up-regulated, while 549 proteins were down-regulated (Fig. 3B). The top five upregulated proteins were identified as ASB6, FABP7, CELF4, LDB1 and PPIG. FABP7 is implicated primarily in neurogenesis and cellular proliferation within the forebrain. LDBI play a pivotal role in retinal development, underlining its significance in visual system development. Conversely, the proteins exhibiting the most significant downregulation included GBB2, K22E, K2C1, K1C10 and ATPMK. Among these, K1C10 plays a crucial role in facilitating keratinocyte migration, which is linked to scar formation, serving as a mechanical barrier to axonal growth (Supplementary Data 1).

In the context of Reg-LI versus control, 835 DEPs were detected. Out of these, 749 proteins were found to be significantly up-regulated, while 86 proteins exhibited a significant downregulation (Fig. 3C). In the LI group, the foremost upregulated proteins were identified as CELF4, TBB4B, PPIG, TTHY and WBP2. TBB4B is integral to the organization of the microtubule cytoskeleton. In contrast, the proteins that demonstrated the most pronounced downregulation included K2C1, RAP1A, COX1, K1C42 and RL37A. Among these, COX1 is implicated in a variety of neurodegenerative diseases (Supplementary Data 2).

In the Deg group, a total of 628 DEPs were found as compared to the control retina. Among these DEPs, 565 proteins were substantially more abundant, while 63 proteins were less abundant (Fig. 3D). Among these, the top five upregulated proteins were identified as TBB4B, TTHY, ESPN, CELF4 and HPCL4. ESPN is implicated in the formation of the actin filament network, playing a critical role in cellular structure and motility. HPCL4 is pivotal in signal transduction processes, mediating the transmission of molecular signals into cellular responses. Conversely, the proteins exhibiting the most significant downregulation included RAP1A, GBB2, COX5B, PTH2 and MGST3. Among these, COX5B is implicated in mitochondrial ATP synthesis coupled proton transport. PTH2 plays crucial roles in regulation of anoikis, a form of programmed cell death (Supplementary Data 3).

### Pathway analysis in Reg-P14 group

**Fig. 4:**
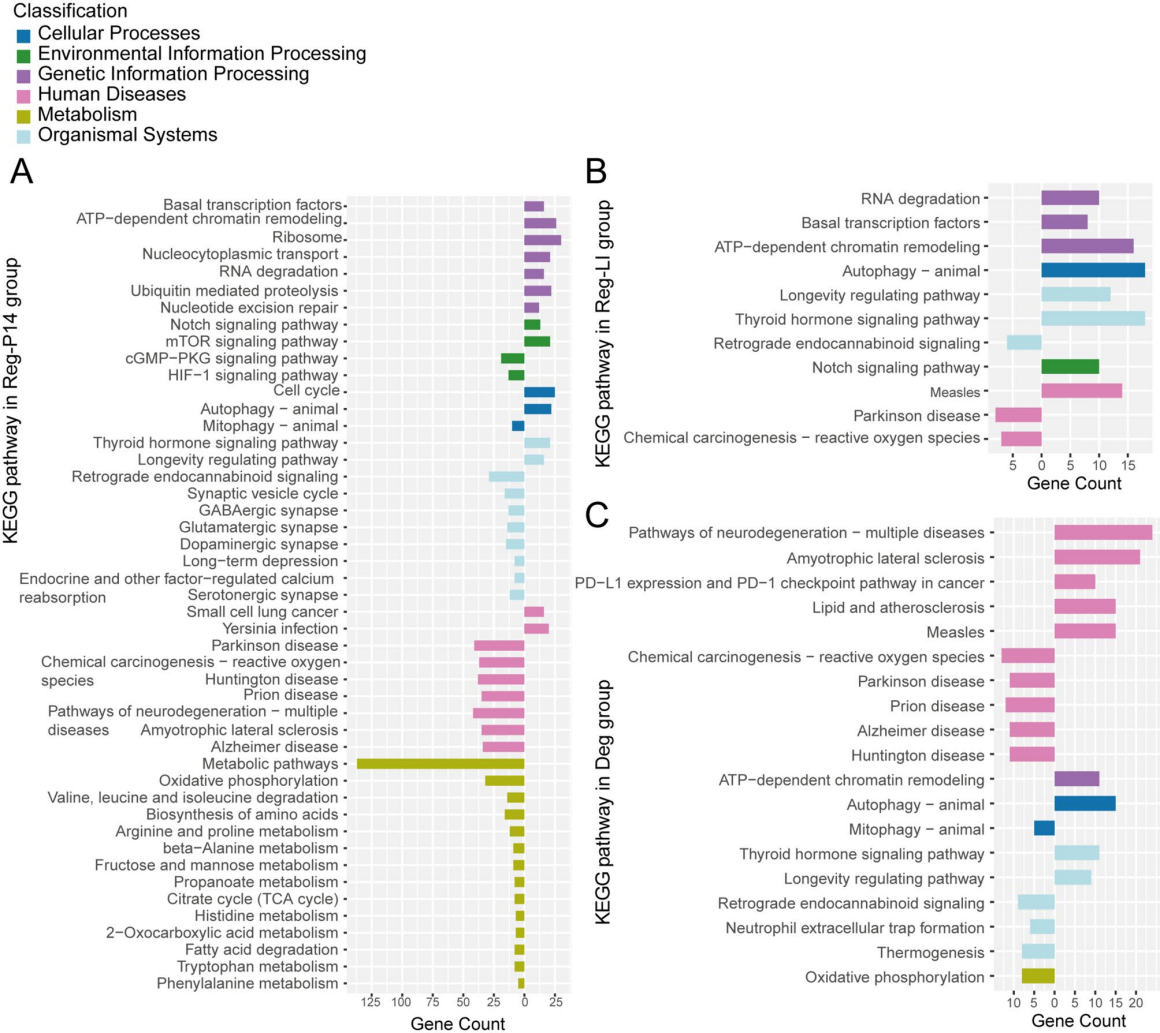
KEGG Pathway Classification Map in (**A**) Postnatal Day 14 (Reg-P14), (**B**) Lens injury (Reg-LI), and (**C**) Degeneration (Deg) Groups. Pathways were classified into six distinct branches according to the classification system of the KEGG database. These branches include cellular processes; environmental information processing; genetic information processing; human diseases; metabolism; organismal systems. Right directions signify pathways enriched with upregulated differentially expressed proteins (DEPs), while left directions indicate pathways characterized by downregulated DEPs.

KEGG pathway analysis in the Reg-P14 group identified significant enrichments in 17 pathways for proteins with increased expression and 54 pathways for those with decreased expression (Fig. 4A). Upregulated DEPs were primarily observed in genetic information processing pathways, environmental information processing pathways and organismal system were affected. The most significantly pathways from upregulated DEPs were basal transcription factors, ATP-dependent chromatin remodeling and ribosome. Downregulated DEPs predominantly impacted metabolic processes, followed by pathways implicated in human diseases and organismal systems. The most significant pathways from downregulated DEPs were metabolic pathways, oxidative phosphorylation and carbon metabolism.

### Pathway analysis in Reg-LI group

KEGG pathway analysis in Reg-LI group revealed significant enrichments in eight pathways for upregulated DEPs and 3 pathways for those downregulated (Fig. 4B). Upregulated DEPs were predominantly observed in pathways related to genetic information processing. Furthermore, analysis of these upregulated DEPs revealed substantial alterations across various biological systems, including organismal systems, cellular processes, environmental information processing pathways, and pathways implicated in human diseases. The most significant pathways from upregulated DEPs were thyroid hormone signaling pathway, ATP-dependent chromatin remodeling and autophagy—animal. Conversely, the most significant pathways from downregulated DEPs predominantly impacted human diseases and organismal systems. The most significant pathway from downregulated DEPs was Parkinson disease pathway.

### Pathway analysis in deg group

KEGG pathway analysis in Deg group identified significant enrichments in nine pathways for upregulated DEPs and 14 pathways for downregulated DEPs (Fig. 4C). Upregulated DEPs were primarily observed in pathways associated with human diseases. Additionally, pathways related to cellular processes, organismal systems and genetic information processing were significantly affected. The most significant pathways from upregulated DEPs included measles, autophagy in animals and PD-L1 expression and the PD-1 checkpoint pathway in cancer. Conversely, pathways from downregulated DEPs predominantly impacted human diseases, followed by pathways implicated in organismal systems, metabolism and cellular processes. The most significant pathways from downregulated DEPs were chemical carcinogenesis—reactive oxygen species, diabetic cardiomyopathy and prion disease.

### Comparative analysis between Reg-P14 and Reg-LI groups

**Fig. 5:**
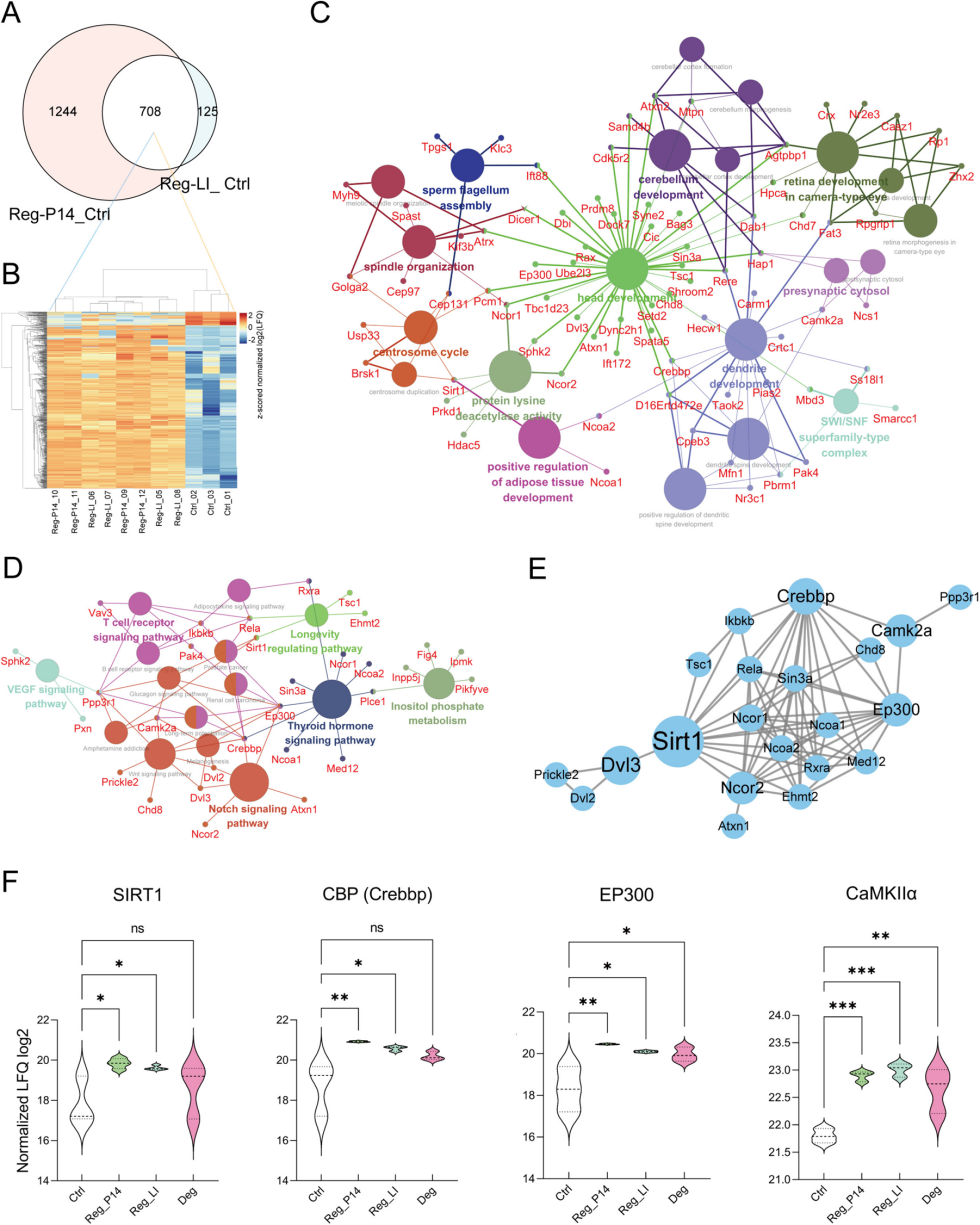
Comparative Analysis between Reg-P14 and Lens Injury (Reg-LI) Groups. (**A**) A Venn diagram illustrating the overlapping and distinct DEPs between the Reg-P14 and Reg-LI groups. (**B**) A cluster map depicting the expression profiles of the 708 proteins commonly regulated in both Reg-P14 and Reg-LI groups, offering a detailed view of their expression patterns. (**C**) Gene Ontology (GO) Term and (**D**) Kyoto Encyclopedia of Genes and Genomes (KEGG) Pathway Analysis from 150 Proteins Associated with Neuro-regeneration. These figures present a clustered visualization of (**C**) GO terms and (**D**) KEGG pathways specifically related to neuro-regeneration, highlighting the proteins involved. Each circle represents a distinct GO term (**C**) or KEGG pathway (**D**), with the size of the circle reflecting the *p* value: larger circles denote lower *p* values, indicating greater statistical significance. Circles filled with the same color represent pathways that share over 50% of their proteins, highlighting significant overlap in protein function within these pathways. (**E**) Protein–Protein Interaction (PPI) network depicting proteins enriched in the pathways shown in (**D**). Circle sizes within this network correlate with the betweenness centrality (BC) values, and larger circle sizes mark higher BC values, indicating greater importance in the protein interaction network. (**E**) Quantitative analyses of log2 label-free quantification (LFQ) intensities across experimental groups, offering quantitative insights into protein abundance variations.

Our research conducted a comprehensive comparative analysis in the Reg-p14 and Reg-LI groups. This investigation identified 708 proteins with shared regulatory patterns across both groups, identifying 1244 proteins uniquely altered in the Reg-P14 group and 125 proteins exclusively regulated in the Reg-LI group (Fig. 5A). The expression profiles of the 708 shared proteins were depicted in a cluster heatmap (Fig. 5B), showcasing expression similarities between the two groups and categorizing these proteins into 653 upregulated and 55 downregulated relative to the control group. The proteins that were most significantly upregulated include CELF4, TTHY and PPIG, while the proteins that were most significantly downregulated comprise K2C1, RAP1A and GBB2.

GO term analysis in these 708 proteins, which exhibit shared regulatory patterns across Reg-P14 and Reg-LI groups, revealed significant enrichments for 223 terms among up-regulated proteins and 4 terms among down-regulated proteins. Notably, there were 150 up-regulated proteins significantly enriched in a cluster including terms such as nervous system development, cell projection organization, neurogenesis and neuron differentiation (Supplementary Data 4). For a more comprehensive understanding of the proteins associated with neuroregeneration, a deeper functional analysis was conducted on the proteins enriched in this term cluster (Fig. 5C). The analysis highlights the biological processes, molecular functions, and cellular components central to neuroregeneration, as deduced from the examined protein set.

To further explore the pathways associated with the 150 neuroregeneration-related proteins enriched in the four aforementioned terms, KEGG pathway enrichment analysis was conducted. The results disclosed enrichments in 15 significant pathways, including thyroid hormone, Notch, Wnt, inositol phosphate metabolism and VEGF signaling pathways (Fig. 5D). In the integrated analysis of pathways and protein–protein interaction (PPI) networks (Fig. 5E), proteins such as SIRT1, CBP (Crebbp), EP300 and CaMKIIα exhibited the most extensive crosstalk among these pathways. Quantitative assessments of log2 LFQ intensities reveal that these proteins are significantly upregulated in the Reg-P14 and Reg-LI experimental groups (Fig. 5F).

### Comparative analysis among Reg-P14, Reg-LI and Deg groups

**Fig. 6:**
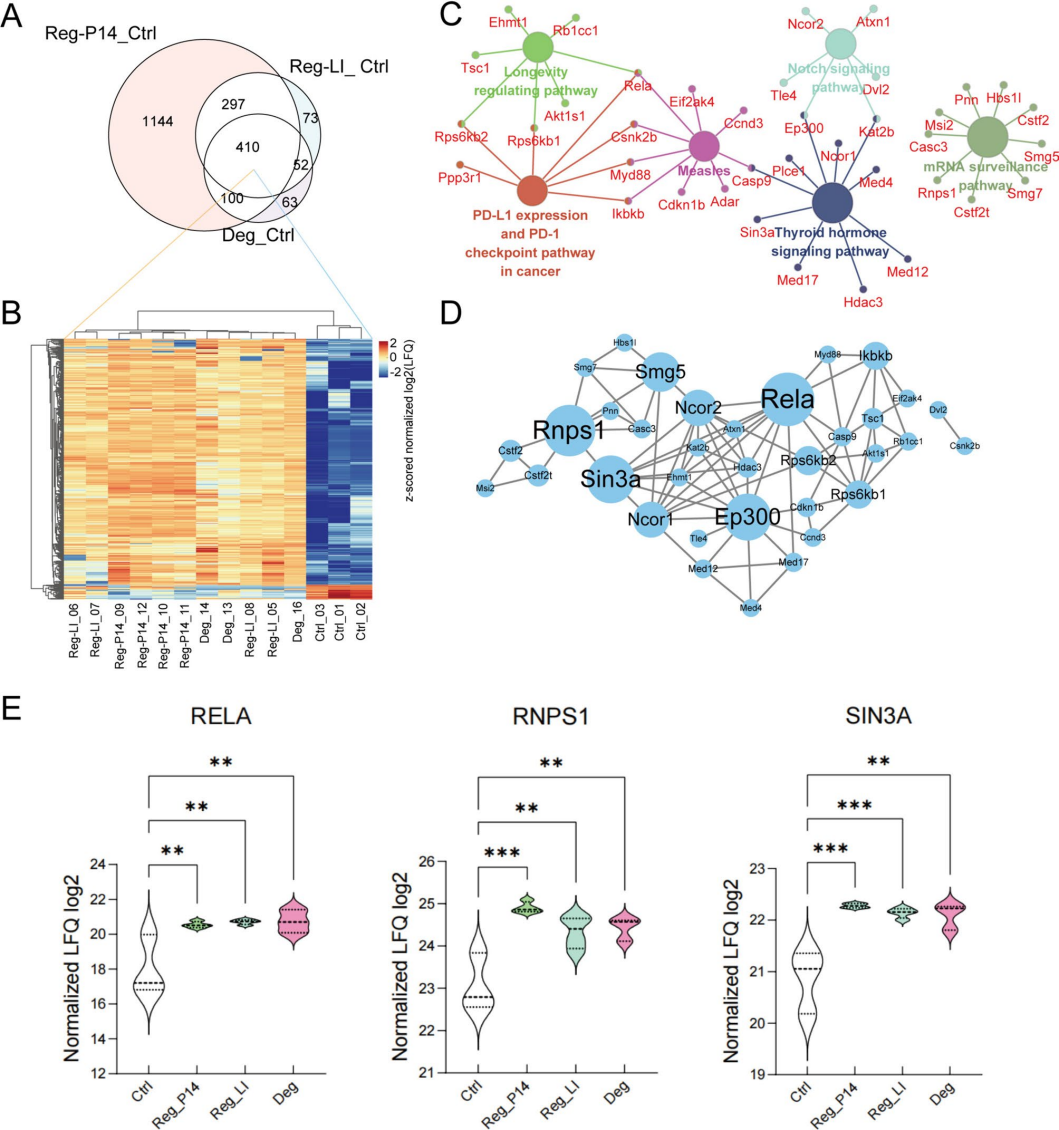
Comparative Analysis among Reg-P14, Lens Injury (Reg-LI) and Degeneration (Deg) Groups. (**A**) A Venn diagram illustrating the shared and unique DEPs among Reg-P14, Reg-LI and Deg groups. (**B**) A cluster map depicting the expression profiles of the 411 proteins commonly regulated in Reg-P14, Reg-LI and Deg groups, offering a detailed view of their expression patterns. (**C**) A clustered visualization of Kyoto Encyclopedia of Genes and Genomes (KEGG) pathways from 411 proteins with shared regulatory patterns across Reg-P14, Reg-LI and Deg groups. Each circle represents a distinct KEGG pathway, with the size of the circle reflecting the *p* value: larger circles denote lower *p* values, indicating greater statistical significance. Circles filled with the same color represent pathways that share over 50% of their proteins, highlighting significant overlap in protein function within these pathways. (**D**) Protein–Protein Interaction (PPI) network depicting proteins enriched in the pathways shown in (**C**). Circle sizes within this network correlate with the betweenness centrality (BC) values, and larger circle sizes mark higher BC values, indicating greater importance in the protein interaction network. (**E**) Quantitative analyses of log2 label-free quantification (LFQ) intensities across experimental groups, offering quantitative insights into protein abundance variations.

Our study conducted a comparative analysis among the Reg-P14, Reg-LI and Deg group. This analysis identified 410 proteins with shared regulatory patterns across all groups (Fig. 6A). The expression profiles of these shared proteins were depicted in a cluster heatmap (Fig. 6B), demonstrating expression parallels cross all groups. This heatmap sorted these shared proteins into 387 upregulated and 23 downregulated compared to the control group. The most significantly upregulated proteins included CELF4, PPIG, TTHY and WBP2 while the most downregulated were RAP1A, GBB2, MGST3 and K2C5.

The GO term analysis on 410 proteins, showing shared regulatory patterns among Reg-P14, Reg-LI and Deg groups, highlighted 102 terms linked to up-regulated proteins and 2 to down-regulated ones. Further, KEGG pathway enrichment analysis identified six significant pathways, including thyroid hormone, Notch, mRNA surveillance and measles signaling pathways, along with PD-L1 expression and the PD-1 checkpoint pathway in cancer (Fig. 6C). Integrated analysis of pathways and PPI networks (Fig. 6D) pinpointed key proteins such as RELA, RNPS1, EP300 and SIN3A for their crucial interactions within this network. The quantification of LFQ log2 show that these proteins are significantly up-regulated in both Reg and Deg groups (Fig. 6E).

## Discussion

The current study took advantage of the state-of-the-art DIA-MS technique to first, comprehensively map the whole proteome of the regenerative retina from p14 mouse pups and adult mice after LI, adult mice, and degenerative retina from adult mice subjected to 2 weeks of elevated IOP. We employed the DAVID Bioinformatics Resources and STRING database to first identify the intercellular communication and pathways lost during development, leading to the irreversible loss of intrinsic axon regeneration ability in RGCs. Additionally, we explored the putative regenerative mechanisms activated by LI. Subsequently, in an endeavor to identify potential targets that can provoke neuroregeneration in degenerative conditions, we compared the interactome and signaling pathways in the experimentally degenerative and regenerative states in the adult retina.

### Proteomic characteristic in P14 mouse retina

Compared to the adult retina, the p14 sample showed significant increase in activities related to genetic information processing, cell cycle, autophagy, and specific signaling pathways like mTOR and Notch. Additionally, there was an upregulation in pathways associated with thyroid hormones and longevity. On the other hand, there was a significant decrease in metabolic activities and pathways linked to neurodegenerative diseases in the p14 sample.

Genetic Information Processing and Cell Cycle Regulation: The increase in activities related to genetic information processing and cell cycle in the p14 retina suggests a critical period of cellular proliferation and differentiation essential for retinal development. This is in line with the notion that the retina undergoes substantial growth and maturation postnatally, requiring extensive transcriptional and translational activities to produce the diverse cellular architecture characteristic of a functional retina^8,9^.

Autophagy, mTOR, and Notch Pathways: The analysis also revealed an upregulation of autophagy and the activation of mTOR and Notch signaling pathways in the p14 retina. Autophagy plays a dual role in removing damaged cellular components, thereby maintaining cellular health, and in modulating differentiation and development^10^. The literature, including studies by Vazquez et al., has documented autophagy's role in neurogenesis within the olfactory bulb^11^. The enhancement of the mTOR pathway aligns with its known functions in promoting cell growth and survival during retinal development^12^. This suggests mTOR pathway may foster axon regeneration and RGC survival in the mature retina following injury, echoing the findings of Eriksen et al. on mammalian retinal regeneration^13^. Similarly, the activation of the Notch pathway, a key regulator of cell fate decisions, underscores its importance in retinal cell differentiation and the establishment of a functional retinal circuitry^14,15^. These mechanisms collectively support a framework wherein the postnatal retina orchestrates cellular proliferation, survival, and differentiation, a requisite for both developmental processes and potential regenerative responses.

Thyroid Hormones and Longevity Pathways: The observed increase in pathways related to thyroid hormones and longevity in the p14 sample points to a regulatory role of thyroid hormones in retinal maturation and a possible enhancement of cellular defense mechanisms during this critical developmental window. This finding is consistent with the work of Arbogast et al. and Sawant et al., which suggested that thyroid hormones significantly influence retinal development and differentiation^16,17^. Additionally, the activation of longevity pathways might indicate a stress resistance phenotype in the developing retina, potentially contributing to cell survival and function^18^.

Metabolic Shifts and Neurodegenerative Pathways: The observed decrease in metabolic activities could be attributed to the developmental hypoxia period, a consequence of an unestablished and dysfunctional capillary network^19^. This phenomenon might also suggest a potential metabolic adaptation and neuroprotective response to postnatal hypoxia^20^. The down-regulation of neurodegenerative pathways may reflect an inherent protective mechanism against the onset of such conditions within these regenerative environments.

### Regenerative mechanisms activated by LI

The comparative analysis between the p14 and LI groups revealed the reactivation of crucial signaling cascades by LI in the mature retina, with the thyroid hormone, Notch and Wnt pathways being particularly emphasized for their critical contributions to neural tissue repair and regeneration. Proteins such as CaMKIIα, CBP and EP300 exhibited the most extensive crosstalk among these pathways.

The thyroid hormone pathway is critical in regulating metabolic processes and neuroplasticity, thus enhancing neural regeneration by optimizing both the metabolic environment and transcriptional activities essential for tissue repair^21,22^. This pathway's influence in axonal regeneration in the mature retina and nervous system aligns with findings from previous studies^23–25^. The Notch and Wnt pathways, both intricate and evolutionarily conserved, govern a variety of cellular functions crucial for neuroregeneration^26–28^. Particularly, the Wnt pathway plays an essential role in the proliferation and differentiation of stem cells, key processes for initiating and sustaining neural tissue regeneration^29^. CaMKIIα, which involved in the Wnt signaling pathway, is essential for calcium signaling within neurons. It's involved in a wide range of cellular functions, including synaptic plasticity, neurotransmitter release, and gene expression^30^. Activation of the CaMKII/CBP axis has been shown to protect long-distance RGC axon projections in vivo, maintaining visual function from the retina to the visual cortex and thus positively influencing visually guided behavior^31,32^. CBP and EP300, implicated in all three pathways, are histone acetyltransferases vital for chromatin remodeling and gene expression regulation, facilitating the transcription of genes required for cell growth, differentiation, and repair^33,34^. In the realm of neuroregeneration, CBP and EP300 are crucial for activating transcription of genes linked to cell survival, neuroplasticity, and axonal growth, supporting regenerative processes in both spontaneous and experimental contexts^35^.

The integration of these pathways reveals a sophisticated regulatory network essential for activating the endogenous mechanisms of neuroregeneration. Targeting these pathways could lead to promising therapeutic strategies for enhancing neuroregeneration.

### Regenerative mechanisms under degenerative conditions

The comparative analysis between the Reg-P14, Reg-LI and Deg groups depicted various pathways activated in both regenerative and degenerative conditions, notably the thyroid hormone, Notch, mRNA surveillance, and measles signaling pathways, along with PD-L1 expression and the PD-1 checkpoint pathway. Protein EP300, SIN3A, RELA and RNPS1 have been identified as pivotal interactors mediating these pathways.

The activation of the thyroid hormone and Notch signaling pathways in both spontaneously regenerative retinas from p14 mice and experimentally induced regenerative mature retinas by LI—and now observed in degenerative conditions—reaffirms the indispensable roles these pathways play in retinal regeneration under both physiological and pathological conditions. This consistency with previous findings, which documented the upregulation of these pathways in contexts of neural degeneration^24,36^, underscores their importance in the regenerative process. EP300 is implicated in both the thyroid hormone and Notch pathways, acting as a transcriptional co-activator with a key role in chromatin remodeling and gene expression regulation^37^. Transcriptional corepressor SIN3A, involved in thyroid hormone pathway, is responsible for the recruiting HDACs to form an HDAC-associated transcriptional complex to regulate the transcription of specific genes^38^. It has been proved that Sin3A/HDAC is critical for mediating stage-specific neuronal gene expression and the sequential stages of neurogenesis^39^. Their presence across different regenerative states indicates their essential function in facilitating the transcriptional responses necessary for cell proliferation, differentiation, and survival.

The mRNA surveillance pathway is crucial for maintaining the fidelity of gene expression, a fundamental process for ensuring the correct synthesis and function of proteins required during the repair and regeneration of neural tissues^40^. RNPS1 (RNA-binding protein with serine-rich domain 1), which plays an important role in this pathway, mediates the increase of mRNA abundance and translational efficiency^41^, and provide protection from ischemic brain injury while inhibiting neuronal death^42^.

The roles of the measles signaling pathway, alongside PD-L1 expression and the PD-1 checkpoint pathway in neuroregeneration require further investigation. Their emerging significance in modulating immune system interactions with the nervous system introduces a novel aspect of neural repair mechanisms^43,44^. Specifically, the PD-1/PD-L1 axis may play a crucial role in attenuating neuroinflammation, potentially creating a more favorable environment for neural regeneration^45^. RELA (transcription factor p65), a subunit of the NF-κB complex, is implicated in both of the measles and PD-L1 pathways, mediating inflammatory responses and apoptosis^46^. Reparative inflammation plays a key role in the early regenerative process with an important contribution of NF-κB signaling in initiating basal cell neurogenesis. Ablation of NF-κB signaling in regenerating neuron cells results in increased neuronal apoptosis^47^. The identification of RELA in both regenerative and degenerative retina underscores its role in modulating inflammation and apoptosis during neural repair, highlighting its potential as a therapeutic target for enhancing regenerative outcomes.

In conclusion, this study depicted the complex proteomic landscape of the regenerative and degenerative retina, unveiling novel insights into the molecular drivers of retinal regeneration. Identifying key proteins and pathways, we highlight potential therapeutic targets that hold promise for revolutionizing treatments for retinal degenerative diseases. Our advancing knowledge paves the way for novel strategies to combat neurodegenerative conditions, promising a brighter future for regenerative medicine and vision restoration.

## Methods

### Animals

In this study, p14 C57BL/6 J mouse pups (n = 4) and male C57BL/6 J mice (n = 4) at the age of 8–10 weeks and a weight of 28–32 g were used. In total four types of retinal samples were used in this study. To mimic the degenerative condition in the retina, a mouse model of chronically elevated intraocular pressure (IOP), induced through the episcleral vein cauterization (EVC), was used^48^. Progressive RGC degeneration was observed^49^. Furthermore, two types of regenerative retinas were used: retinas from p14 mice which are spontaneously regenerative^50^, and retinas from adult mice subjected to lens injury (LI), which has been proven to promote the regeneration of RGC axons in mature retinas^51^. Untreated retinas from adult mice served as controls. Table 1 gives an overview over the different experimental procedures.

**Table 1:** Overview of the different groups and their outcomes.

Groups	Control	p14	LI	EVC
Animals	Male C57BL/6J mice (n = 4)	p14 C57BL/6J pups (n = 4)	Male C57BL/6J mice (n = 4)	Male C57BL/6J mice (n = 4)
Intervention	None	None	Lens injury	Elevated IOP induced by episcleral vein cauterization (EVC)
Follow-up	N/A	N/A	2 days	14 days
Cultured under regenerative conditions	7 days in culture	7 days in culture	7 days in culture	7 days in culture
Types of retinas	Non-regenerative	Spontaneously regenerative	Experimentally regenerative	Degenerative
Immunohistochemistry	Anti-Brn3a	Anti-beta-III-tubulin	Anti-beta-III-tubulin; Anti-Brn3a	Anti-Brn3a
DIA-MS	Yes	Yes	Yes	Yes

Animal experiments were approved by the State Office for Nature, Environment and Consumer Protection North Rhine-Westphalia (LANUV Landesamt für Natur, Umwelt und Verbraucherschutz Nordrhein-Westfalen, permission number: 81-02.04.2020. A490; approval date: 25.03.2021). All experimental procedures adhered to the guidelines outlined in the Association for Research in Vision and Ophthalmology (ARVO) Statement for the Use of Animals in Ophthalmic and Vision Research, as well as the protocols established by the Institutional Animal Care and Use Committee. Our study is reported in accordance with Animal Research: Reporting of In Vivo Experiments (ARRIVE) guidelines. The animals were housed within the Animal Facilities of the Medical Faculty at the University of Cologne with a 12-h day-night cycle. Water and food were facilitated ad libitum.

Surgical procedures were conducted under general anesthesia, administered through intraperitoneal injection. The anesthetic protocol comprised a combination of 0.50 ml Xylazinehydrochloride (Xylazine, Dutch Farm International, Germany) and 1.00 ml Ketamine (Ketamine Inresa, Inresa Arzneimittel GmbH, Germany), diluted in 8.5 ml of sodium chloride. The dosage was calibrated at 0.1 ml per 10 g of body weight. Oxybuprocaine hydrochloride (Novesine, OmniVision, Puchheim, Germany) was applied topically to the ocular surfaces before the intervention. All animals were observed directly by the staff at the animal facilities of the medical faculty of Cologne after each surgery and once a day until euthanasia.

### *Lens* injury (LI) to induce regeneration of RGC axons in mature retina

Adult male C57BL6J mice (n = 4) were anesthetized as described above. Lens injury was performed in the right eye while the left eye served as control. After administration pupil dilatation with mydriatic (Tropicamide, PZN-01875775), LI was induced by puncturing the lens capsule using a 30G needle via a retro-lenticular approach as described before^51^. The tip of the needle was bent at a 90° angle and inserted into the eye perpendicular to the sclera so as to intentionally puncture the lens capsule.

### Episcleral vein cauterization (EVC) to induce elevated IOP

Chronically elevated IOP was induced through the cauterization of three episcleral veins in the right eyes of the adult male C57BL6J mice (n = 4) and the left eyes served as control, following previously established protocols^52^. In brief, following anesthesia, an incision was made on the conjunctiva and Tenon's capsule to expose the episcleral veins. The major trunks of these veins were then cauterized using an ophthalmic small vessel cauterizer (Fine Science Tools GmbH, 18000-00, Germany). Subsequently, the conjunctiva was carefully readjusted and ofloxacin ointment was given onto the ocular surface to prevent infection. To assess IOP changes, measurements were taken before and right after the surgery and at intervals at day 1, day 2, day 7 and day 14 post-surgery, using a TonoLab rebound tonometer (iCare, Vantaa, Finland) on conscious animals^53,54^.

### Preparation of retinal explants

The adult mice and the p14 pups were euthanized via cervical dislocation. Eyes were enucleated immediately post-mortem and transferred to a petri dish containing ice-cold betaisadona solution (Braunol, Braun, Germany) for 3 min and medium was then changed into ice-cold sterile Hank’s Balanced Salt Solution (HBSS; Gibco BRL, Eggenstein, Germany). The anterior segment of the eye was detached and the retina uncovered. The intact retina was separated from the optic cup and the vitreous body was removed. Explants were placed with the ganglion cell layer up on Millipore filters (A045R047Z-P, Japan). The retinal explants from adult mice were carefully dissected into 4 quarters for further experiments or analysis.

### Culture under regenerative conditions

Retinas from P14, LI, EVC and control groups were carefully dissected and whole-mounted on mixed cellulose filters (A045R047Z-P, Japan). Each whole-mounted retina was divided radially into four sections using a McIlwain tissue chopper. Subsequently, one quarter of the retina was further segmented radially into two parts using the same instrument, each part was cultured in a lumox culture dish 50 (94.6077.410) pre-coated with poly-d-lysine and laminin, as described in detail in our previous study^55^. Serum-free S4 growth medium as described in our previous study was used^55^. The retinal explants were positioned with the ganglion cell layer facing down. These explants were incubated at 37 °C in an atmosphere of 5% CO_2_ and 55% O_2_ for a duration of 7 days.

### Quantification of RGCs

In total three retinal quarters per group were used for RGC quantification to validate neurodegeneration after EVC. RGCs in retinal explants were labeled by Brn3a immunohistochemical staining as previously described^56,57^. Briefly, the retinal explants were washed in phosphate-buffered saline (PBS), fixed in 4% paraformaldehyde (PFA; pH = 7.4), and then in 30% sucrose solution overnight. Non-specific binding was blocked with 1% milk powder and 0.3% Triton in PBS. The tissues were subsequently incubated with primary antibody overnight at 4 °C, followed by washing, and then exposed to Alexa-Fluor-488-conjugated secondary antibody (1:1000) for 2 h in darkness. The explants were then washed and mounted using antifade mounting media with 4′6-Diamidino-2-phenylindol (DAPI, VEC-H-1200). Fluorescence-positive RGCs were visualized and photographed with a fluorescent microscope under 20-fold magnification (Imager.M2 ApoTome.2 Carl Zeiss, Germany, 4 pictures per retinal quarter). Total numbers of RGCs were counted using ImageJ (ImageJ Fiji version 1.5), An average value per retinal quarter was calculated and then converted to RGCs/mm^2^. Mean values were compared between groups. Primary antibodies used were mouse anti-brain-specific homeobox/POU domain protein 3A (Brn-3a) monoclonal antibody (1:200; EMD Millipore Corp., MAB1585, USA). Secondary antibodies used were goat anti-mouse conjugated with Alexa Flour 488 (1:1000; abcam, ab150113).

### Immunohistochemistry of RGC axons

After 7 days in culture, outgrowing neurites have been visualized and photographed natively with a binocular microscope (n = 8) with 40-fold magnification. On day 7 post explantation, neurites were identified immunohistologically by anti-β-III-Tubulin (Purified anti-Tubulin β 3, BioLegend). In brief the retinal explants with their neurites were fixed inside of the petri dishes in 4%-formalin-solution, blocked in 2% bovine serum albumin, 0.3% Triton-X and 5% goat serum for 60 min. The β-III-Tubulin-AB was diluted in blocking solution and incubated overnight at 4 °C. Following neurites were visualized with a fluorescent microscope (Nikon Eclipse TS100, Germany) using 20-fold magnification. Under usage of Image J neurites have been counted. Subsequently, mean values of the particular experimental groups were calculated and compared. Images were processed and presented using GNU Image Manipulation Program (GIMP 2.10.34).

### Sample preparation for mass spectrometry

Retinal proteins were extracted by tissue protein extraction reagent (T-PER, Thermo Scientific, MA, USA) as described previously by Manicam et al.^58^. Homogenization of the tissue was achieved using a tissue squasher. Subsequently, the sample was subjected to centrifugation at 10,000*g for a duration of 5 min, resulting in the pelleting of tissue debris. The supernatant was then carefully collected, and the protein concentration for each sample was determined using a standard bicinchoninic acid (BCA) protein assay kit (Pierce, Rockford, IL, USA). This quantification procedure was executed in accordance with the manufacturer's instructions. Samples with a protein concentration exceeding 1 μg/μL underwent further processing via SP3 sample preparation. In detail, the SP3 preparation process involved adding an equal volume of 2*SP3 lysis buffer, consisting of 10% SDS in PBS, to the respective samples. The resultant mixture was homogenized through pipetting and subsequently subjected to a heat treatment at 95℃ for 5 min. To facilitate reduction, 100 mM Dithiothreitol (DTT) was added to each sample, achieving a final concentration of 5 mM. Subsequent to vortexing, the samples were incubated at 55℃ for a duration of 30 min. The alkylation process involved the addition of 400 mM Chloroacetamide (CAA), reaching a final concentration of 40 mM. Following thorough vortexing, the samples were incubated in the dark at room temperature for 30 min, shielded by tin foil. Then the samples were once again subjected to centrifugation at 20,000*g for a period of 10 min. Subsequently, the supernatant was carefully transferred to new tubes. Finally, to ensure sample preservation, they were frozen at − 20 ℃ and subsequently submitted to the CECAD/CMMC proteomics core facility at the University of Cologne for further DIA-MS.

### Mass spectrometry analysis

Samples were analyzed by the Cologne Excellence Cluster for Aging and Aging-related Diseases (CECAD Proteomics Facility, Cologne, Germany) on an Orbitrap Exploris 480 (Thermo Scientific, granted by the German Research Foundation under INST 1856/71-1 FUGG) mass spectrometer equipped with a FAIMSpro differential ion mobility device that was coupled to an UltiMate 3000 (Thermo Scientific). Samples were loaded onto a precolumn (Acclaim 5 µm PepMap 300 µ Cartridge) for 2 min at 15 ul flow before reverse-flushed onto an in-house packed analytical column (30 cm length, 75 µm inner diameter, filled with 2.7 µm Poroshell EC120 C18, Agilent). Peptides were chromatographically separated at a constant flow rate of 300 nL/min and the following gradient: initial 6% B (0.1% formic acid in 80% acetonitrile), up to 32% B in 72 min, up to 55% B within 7.0 min and up to 95% solvent B within 2.0 min, followed by column wash with 95% solvent B and reequilibration to initial condition. The FAIMS pro was operated at − 50 V compensation voltage and electrode temperatures of 99.5 °C for the inner and 85 °C for the outer electrode.

For the gas-phase fractionated library, a pool generated from all samples was analyzed in six individual runs covering the range from 400 m/z to 1000 m/z in 100 m/z increments. For each run, MS1 was acquired at 60 k resolution with a maximum injection time of 98 ms and an AGC target of 100%. MS2 spectra were acquired at 30 k resolution with a maximum injection time of 60 ms. Spectra were acquired in staggered 4 m/z windows, resulting in nominal 2 m/z windows after deconvolution using ProteoWizard^59^.

For the samples, MS1 scans were acquired from 399 to 1001 m/z at 15 k resolution. Maximum injection time was set to 22 ms and the AGC target to 100%. MS2 scans ranged from 400 to 1000 m/z and were acquired at 15 k resolution with a maximum injection time of 22 ms and an AGC target of 100%. DIA scans covering the precursor range from 400 to 1000 m/z and were acquired in 60 × 10 m/z windows with an overlap of 1 m/z. All scans were stored as centroid.

The gas-phase fractionated library was build using DIA-NN 1.8.1@@^60^ using A Swissprot mouse canonical database (UP589, downloaded 04/01/22) with settings matching acquisition parameters. Samples were analyzed in DIA-NN 1.8.1 as well using the previously generated library and identical database. DIA-NN was run with the additional command line prompts “—report-lib-info” and “—relaxed-prot-inf”. Further output settings were: filtered at 0.01 FDR, N-terminal methionine excision enabled, maximum number of missed cleavages set to 1, min peptide length set to 7, max peptide length set to 30, min precursor m/z set to 400, max precursor m/z set to 1000, cysteine carbamidomethylation enabled as a fixed modification. Afterwards, DIA-NN output was further filtered on library q-value and global q-value ≤ 0.01 and at least two unique peptides per protein using R (4.1.3). Finally, label-free quantification (LFQ) values calculated using the DIA-NN R-package. Afterwards, analysis of results was performed in Perseus 1.6.15@@^61^. Normalized LFQ data from DIA-NN for proteins with at least two unique peptides were log2 transformed and samples checked for data quality based on identified proteins and two samples were removed due to low protein identifications. Afterwards, proteins IDs were filtered for data completeness removing all proteins not present in all samples of at least one replicate group and remaining proteins annotated for GO, KEGG, Pfam and Reactome pathway names. Imputation was performed using the MinDet algorithm from the ImputeLCMD package embedded into the Perseus R plugin^62^. Finally, one-way ANOVA and T-tests (Student’s or Welch’s, depending on even or uneven replicate numbers in specific comparison) were performed to identify significantly changed proteins. All tests were FDR-based with a cutoff of 0.05 and s0 of 0.2. All detected proteins were Z-scored and used for hierarchical clustering with standard conditions.

### Enrichment analysis

The identification and visualization of differentially expressed proteins (DEPs) were executed using Python code. Significance was determined based on a cutoff value of FDR adjusted *p* value ≤ 0.05, and differential expression was defined as a log2 Fold change > 1 or < − 1. Gene Ontology (GO) term and Kyoto Encyclopedia of Genes and Genomes (KEGG) pathway enrichment analyses were conducted employing DAVID Bioinformatics Resources (https://david.ncifcrf.gov/tools.jsp)@@^63,64^ and KEGG database (https://www.kegg.jp)@@^65–67^. Statistical significance was defined as FDR < 0.05. Protein–protein interactions were undertaken with the String database (https://string-db.org/). ClueGO and CytoNCA plugins within Cytoscape were employed for the graphical representation of enrichment outcomes.

### Statistical analysis

Statistical analyses for RGC quantification and IOP, along with figure presentation, were conducted using Prism 8 software (GraphPad Software, Inc., San Diego, CA, USA). The data are presented as mean ± standard deviation (SD). The assessment of significant differences between groups was performed using either a one-way analysis of variance (ANOVA) or an unpaired t-test. Statistical significance was defined as *p* < 0.05.

**Fig. 7:**
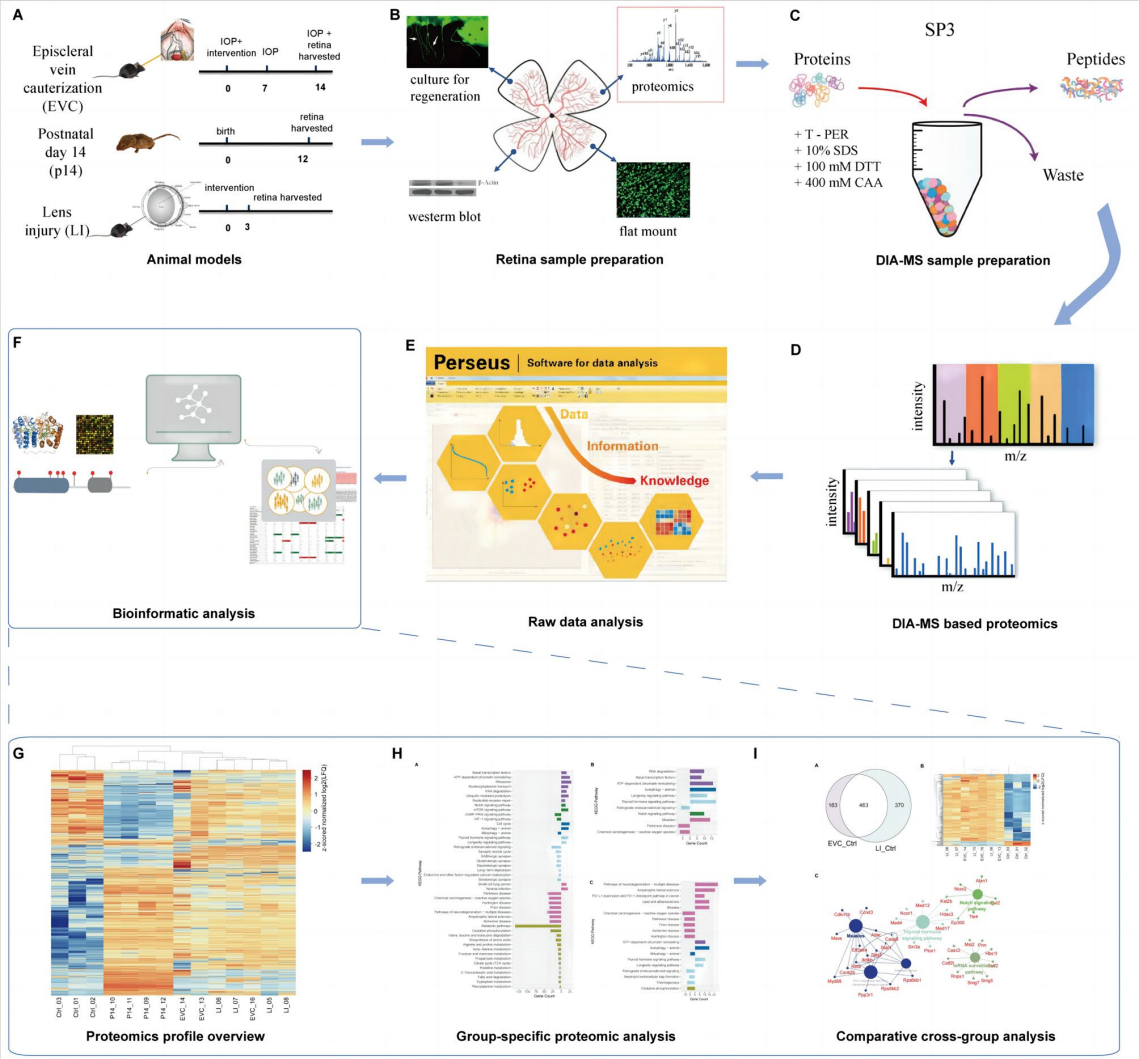
Experimental Workflow Overview. (**A**) Animal model establishment: Glaucomatous retinal ganglion cell (RGC) degeneration models were induced in 9-week-old C57BL6J mice through episcleral vein cauterization (EVC). Experimental RGC regeneration was initiated by lens injury (LI) in 9-week-old C57BL6J mice. Mice were euthanized at specific time points: 2 weeks post-EVC surgeries, 14 days post-birth (p14), and 2 days after LI. (**B**) Retina harvesting and preparation: After successful animal model establishment, retinas were harvested and prepared for diverse analytical techniques, including proteomic analysis, regeneration culture, and immunohistochemistry (IHC). (**C**) DIA-MS sample preparation (SP3 Protocol): Retinal samples underwent preparation for DIA-MS using the Single-Pot Solid-Phase-enhanced Sample Preparation (SP3) protocol. (**D**,**E**) DIA-MS-based proteomic analysis: Prepared samples underwent comprehensive DIA-MS-based proteomic analysis to profile the retinal proteome, allowing quantitative assessment of protein abundance and expression. (**F**) Bioinformatic analysis: The acquired proteomic data underwent bioinformatic analyses: (**G**) Proteomics profile overview: This panel presents a comprehensive overview of the proteomic datasets employed for subsequent analytical processes. (**H**) Group-specific proteomic analysis: This section illustrates detailed pathway analyses for the p14, LI, and EVC groups, elucidating the distinct proteomic signatures associated with each experimental condition. (**I**) Comparative cross-group analysis: This analysis compares proteomic profiles across different experimental groups, aiming to pinpoint both unique and shared molecular expressions, pathways, and interactions.

The overview of experimental procedures encompassing animal modeling, sample preparation, and the proteomics workflow employed in this investigation, is presented in Fig. 7.

## Supplementary Information

Supplementary Information.

## Data Availability

The data that support the findings of this study have been deposited in ProteomeXchange with identifier PXD049992. https://www.ebi.ac.uk/pride/profile/reviewer_pxd049992 (Reviewer account details: Username: reviewer_pxd049992@ebi.ac.uk, Password: fou5SAeC).
